# A convolutional neural network for the screening and staging of diabetic retinopathy

**DOI:** 10.1371/journal.pone.0233514

**Published:** 2020-06-22

**Authors:** Mohamed Shaban, Zeliha Ogur, Ali Mahmoud, Andrew Switala, Ahmed Shalaby, Hadil Abu Khalifeh, Mohammed Ghazal, Luay Fraiwan, Guruprasad Giridharan, Harpal Sandhu, Ayman S. El-Baz

**Affiliations:** 1 Electrical and Computer Engineering, University of South Alabama, Mobile, AL, United States of America; 2 Bioengineering Department, University of Louisville, Louisville, KY, United States of America; 3 Abu Dhabi University, Abu Dhabi, UAE; 4 Department of Ophthalmology and Visual Sciences, University of Louisville, Louisville, KY, United States of America; University of Central Florida (UCF), UNITED STATES

## Abstract

Diabetic retinopathy (DR) is a serious retinal disease and is considered as a leading cause of blindness in the world. Ophthalmologists use optical coherence tomography (OCT) and fundus photography for the purpose of assessing the retinal thickness, and structure, in addition to detecting edema, hemorrhage, and scars. Deep learning models are mainly used to analyze OCT or fundus images, extract unique features for each stage of DR and therefore classify images and stage the disease. Throughout this paper, a deep Convolutional Neural Network (CNN) with 18 convolutional layers and 3 fully connected layers is proposed to analyze fundus images and automatically distinguish between controls (i.e. no DR), moderate DR (i.e. a combination of mild and moderate Non Proliferative DR (NPDR)) and severe DR (i.e. a group of severe NPDR, and Proliferative DR (PDR)) with a validation accuracy of 88%-89%, a sensitivity of 87%-89%, a specificity of 94%-95%, and a Quadratic Weighted Kappa Score of 0.91–0.92 when both 5-fold, and 10-fold cross validation methods were used respectively. A prior pre-processing stage was deployed where image resizing and a class-specific data augmentation were used. The proposed approach is considerably accurate in objectively diagnosing and grading diabetic retinopathy, which obviates the need for a retina specialist and expands access to retinal care. This technology enables both early diagnosis and objective tracking of disease progression which may help optimize medical therapy to minimize vision loss.

## I. Introduction

Convolutional neural networks (CNNs) have been recently utilized for diagnosing diabetic retinopathy (DR) through analyzing fundus images and have proven their superiority in detection and classification tasks [1] [2]. For diabetes, DR is a major complication that may eventually result in vision loss as well as blindness. It is caused by the damage occurring to the retina blood vessels as increased levels of blood sugar block minute blood vessels that supply blood to the retina. Almost 171 million individuals worldwide were diagnosed with diabetes in 2000, and it is expected that this number will rise to 366 million by 2030 [3]. DR may have different abnormal effects on the retina e.g., microaneurysms, hard and soft exudates, hemorrhages, neovascularization and macular edema. Furthermore, DR can be classified into five stages, which are mild non-proliferative DR (NPDR), moderate NPDR, severe NPDR, proliferative DR (PDR) and macular edema (ME) [3]. Mild NPDR is the disease earliest stage that may advance to proliferative diabetic retinopathy where the vision loss occurs and the eye is filled with interstitial fluids. At earlier stages, patients are often asymptotic. However, with the disease progression, symptoms can include blurred vision, blind spots, distorted central vision, large floaters and sometimes sudden loss of vision. Hence, it is critical to detect the disease at earlier stages and provide an accurate diagnosis and staging in order to possibly reduce the disease complications and the risk of the vision loss.

Diagnosis of DR is most commonly done by dilated eye examination that is performed by ophthalmologists. Other methods of disease diagnosis include fluorescein angiography, optical coherence tomography (OCT) or fundus photography. For fluorescein angiography, the blood flow and vascular abnormalities are photographed upon the intravenous injection of contrast dye. In OCT, the retinal structure, thickness, and edema (i.e. retinal swelling) are evaluated. Currently, diagnosis of DR is subjective and needs to be performed by a retina specialist that passed a specialized training for diagnosis and grading as the visual assessment and manual measurements of changes in retinal vasculature and layers are deemed very complex tasks. Unfortunately, a lot of diabetic patients attempt to visit a retina specialist only with symptomatic vision loss, when their pathology gets advanced and mostly irreversible, due to inadequate access to trained eye-care professionals and tertiary eye-care services. Based on this, there is a clinically significant motivation to have an objective and non-invasive diagnostic system that is capable of not only accurately detecting DR at an early stage but also grading it.

Machine learning techniques have been used in DR detection and classification [4–14]. Acharya et al. introduced an automated diagnosis method using SVM classifier to identify normal, mild DR, moderate DR, severe DR, and prolific DR [4]. The proposed method was trained on 300 subjects of different disease stages and achieved an accuracy of 82%, sensitivity of 82%, and specificity of 88%. The authors proposed another system where hemorrhages, micro-aneurysms, exudates, and blood vessels were extracted from raw images of 331 subjects and fed to SVM for classification [5]. The system provided a classification accuracy of 85.9%, a sensitivity of 82%, and a specificity of 86%.

Nayak et al. developed a CNN model to identify non-DR, NPDR, and PDR [6]. Morphological processing techniques and texture analysis methods were applied on fundus images of 140 subjects to detect features such as hard exudates and blood vessels. A classification accuracy of 93%, a sensitivity of 90%, and a specificity of 100% were achieved. Pratt et al. proposed a CNN and data augmentation that can identify features such as hemorrhages, micro-aneurysms, and exudates on the retina, and therefore differentiate between the five stages of the disease [7]. The network was trained on a Kaggle dataset of 80,000 fundus images using a graphical processing unit (GPU). The proposed CNN achieved an accuracy, a sensitivity, and a specificity of 75%, 30%, and 95%, respectively. Furthermore, Shaban et al. introduced a CNN trained on 101 fundus images that can accurately identify the four stages of the disease (i.e. non-DR, NPDR, severe NPDR and PDR) [8]. A leave-one-out approach was used for testing. The proposed method attained an accuracy of 80.2%, a sensitivity of 78.7%, and a specificity of 84.6%. Moreover, Dekhil et al. introduced a fine-tuned VGG-16 trained on the public Kaggle dataset [17] classifying subjects with an accuracy of 77% and quadratic weighted kappa score of 78% [9].

Gao et al. created a dataset of DR fundus images and trained a modified version of the Inception v.3 network with the aid of data processing and data augmentation stages. The proposed network achieved an accuracy of 88.72% for classifying the severity of DR into one of four grades [10]. Furthermore, Hu et al. introduced a deep neural network architecture to classify Retinopathy of Prematurity (ROP) disease based on the existence and severity of the disease [11]. The proposed network consists of two subnetworks where the first subnetwork extracts high level features from fundus images, which are fused by an aggregate operator and fed into the second subnetwork for predicting the stage of the disease. The proposed method yielded an improved testing accuracy when the Inception v.2 network was used compared to other standard networks such as VGG-16 and ResNet-50.

Mizutani et al. introduced a computer aided diagnosis (CAD) method to detect micro-aneurysms on retinal fundus images [12]. Jaafar et al. presented an automatic approach for detecting soft and hard exudates considered as the early signs of DR [13]. Morphological operations, filters and thresholds were used to detect macular abnormalities on fundus images for DR diagnosis while the thickness of the retinal nerve fiber layer was determined on OCT images for the diagnosis of glaucoma by Pachiyappan et al. [14]. Tan et al. proposed an algorithm to extract retinal vasculature to obtain and detect blood vessels [15]. However, all these prior methods require image processing and data augmentation, which increases their complexity and complicates adaptation to a clinical setting.

In this paper, we propose a novel deep CNN architecture that can classify subjects with high accuracy into controls (i.e. no DR), moderate DR that includes patients with mild or moderate NPDR, and severe DR, which represents patients in the late stages with either severe NPDR or PDR. The proposed architecture was trained and tested on 4,600 fundus images generated from a public Kaggle dataset of 3,661 images [17]. Five-fold and 10-fold cross-validation methods were used to measure the performance of the proposed architecture including validation accuracy, Quadratic Weighted Kappa Score, sensitivity, specificity, Receiver Operating Characteristic Curve (ROC), and the Area Under Curve (AUC). Confusion matrices were also provided to offer an understanding of the classifier behavior and performance. We have also compared our proposed architecture with the latest state-of-the-art architectures used in DR diagnosis and staging.

## II. Proposed CNN model

### A. Dataset description

Fundus images used in this study are publicly available from Kaggle [17]. Images were provided by the Asia Pacific Tele-Ophthalmology Society (APTOS) as part of the 2019 blindness detection competition. Almost 3,648 high resolution fundus images were selected from the Kaggle dataset of 3,661 images taken by different models and types of cameras in multiple clinics over an extended period of time. Further, images may contain artifacts, be out of focus, underexposed, or overexposed.

Images were scored on a scale of 0 to 4. Table 1 shows the class labels or score, the corresponding DR stage, and class size for the dataset. From Table 1, the dataset is unbalanced with most of the images belong to the first and third classes. In order to induce a more balanced dataset, and to accurately classify images using CNN, we have split the dataset into three categories such that both labels ‘1’ and ‘2’ represent the moderate version of the disease while both ‘3’ and ‘4’ describe the severe DR category. The category labels and sizes are indicated in Table 1 as well.

**Table 1:** APTOS 2019 Kaggle dataset classes description.

Class Label	DR Stage	Class Size	Category Label	Category Size
0	No DR	1,796	0	1,796
1	Mild NPDR	369	1	1,364
2	Moderate NPDR	995		
3	Severe NPDR	193	2	488
4	PDR	295		

**Fig 1:**
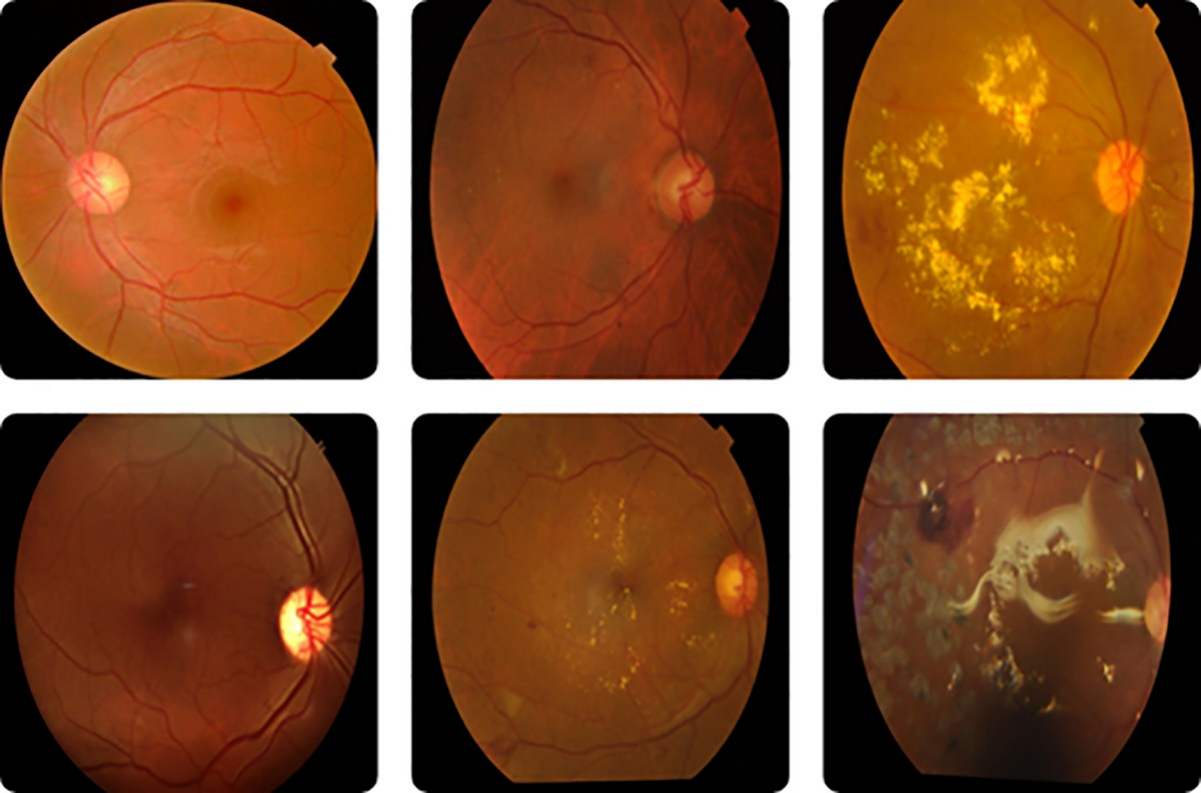
Fundus images for the five stages of DR.

Examples of the fundus images belonging to the dataset are shown in Fig 1. The leftmost column of images belongs to control subjects. The middle column corresponds to mild and moderate NPDR subjects, respectively. The rightmost column belongs to severe NPDR and PDR subjects, respectively.

### B. Proposed model description

CNN is an artificial neural network architecture that aims at learning low and high level features of medical images in an automated manner which helps in the detection, classification, and staging of medical diseases [1] [2]. CNN usually consists of several layers including convolutions, pooling, and fully connected layers. The output of each layer is called an activation or a feature map, which can be an input to another layer. A set of linear filters is applied to the input image or the activation map in the convolutional layer to extract a number of different low or high level features such as edges, curves, blood vessels, etc. The output of a 3×3 convolution is defined as follows:

$$y\left(l,m,n\right)=\sum_{k=1}^{3}\sum_{i=1}^{3}\sum_{j=1}^{3}\begin{array}{c} w\left(l,i,j,k\right)x\left(i+m-1,j+n-1,k\right)+b\left(l\right) \end{array}$$ (1)

where *x(i*,*j*,*k)* ithe image gray level value, and *w(l*,*i*,*j*,*k)* and *b(l)* represent the weights and biases, respectively, of the convolutional layer. The pooling layer is usually used to reduce the number of parameters (i.e. weights and biases) of the network by subsampling the activation maps, as well as improve the robustness of the extracted features. The pooling layer can be realized either using a set of linear filters that computes the average of the pixel values included within a masked area in the image (i.e. average pooling) or using a set of non-linear filters that sorts the pixel values within some area in the image and obtains the maximum (i.e. max pooling). A 2×2 max pooling layer generates robust low dimensional features *z (l*,*m*,*n)* defined as follows:

$$\begin{array}{c} z(l,m+1,n+1)=\\ Max[\begin{array}{cc} y(l,2m+1,2n+1) & y(l,2m+1,2n+2)\\ y(l,2m+2,2n+1) & y(l,2m+2,2n+2) \end{array}] \end{array}$$ (2)

A fully connected layer consists of a set of neurons that are connected with all the activation maps of the neurons of previous layers. The outputs of both convolutional and early fully connected layers are usually processed using a Rectified Linear Unit (ReLU) defined as follows:

$$a_{i}=\{\begin{array}{c} b_{i}\begin{array}{cc} & b_{i}>0 \end{array}\\ 0\begin{array}{cc} & b_{i}<0 \end{array} \end{array}$$ (3)

where *b*_*i*_ is an input to the ReLU and *a*_*i*_ is the corresponding activation generated by the ReLU. However, the soft max activation function is deployed at the end of the network to compute the probability distribution of each of the final fully connected layer outputs as follows:

$$a_{i}=\frac{e^{-c_{i}}}{\sum_{j=0}^{L}e^{-c_{j}}}$$ (4)

where *c*_*i*_ is the *i*^*th*^ output of the last fully connected layer, *L* is the number of classes and *a*_*i*_ is the corresponding SoftMax activation.

The cross entropy loss *e*, which describes the deviation of the predicted outputs of the SoftMax from the expected desired outputs, is defined as follows:

$$e=-\sum_{j=0}^{L}{\overset{⌢}{a}}_{j}\log(a_{j})$$ (5)

where ${\overset{⌢}{a}}_{j}$ is the actual probability (i.e. expected desired probability for a certain fundus image belonging to a certain class at the last fully connected layer *j*^*th*^ output). The cross entropy loss is then minimized using the Stochastic Gradient Descent (SGD) in order to update the model parameters that will allow the successful classification of images. The aforementioned optimization approach is known as the backpropagation algorithm. The Max pooling layer parameters such as the number of filters, filter size, and stride are usually set in advance and, therefore, do not require training.

In this paper, a CNN was introduced to successfully classify DR subjects into non-DR, moderate DR, and severe DR and hence stage the disease in an automated fashion. First, a pre-processor was used to resize fundus images to maintain the same standard size of 224×224×3. Further, a class-specific data augmentation approach was adopted to expand the size of the smallest category with category label “2” in order to improve the performance of the proposed architecture when being applied on the unbalanced dataset. About 480 images were extracted from the smallest category and augmented by 90^o^ and 180^o^ rotation of the images, generating an expanded set of 1440 images that is similar to other categories with respect to size.

**Fig 2:**
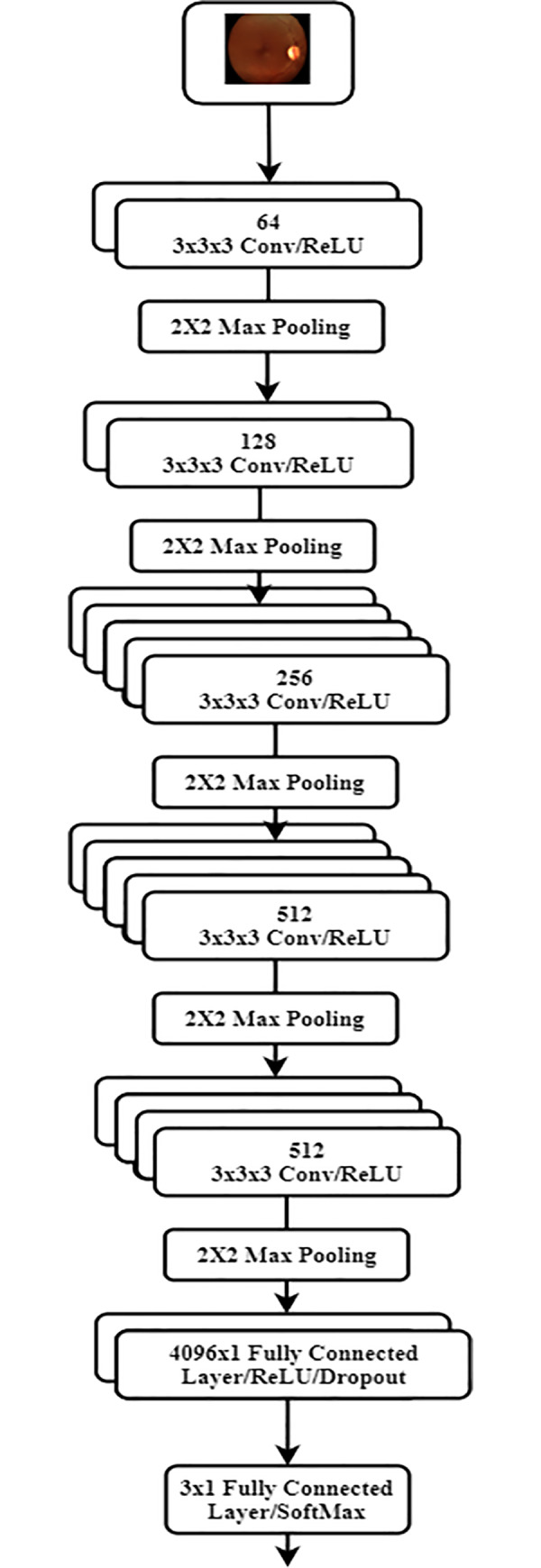
Proposed CNN architecture.

Secondly, fundus images were provided to a set of five consecutive stages of convolutional layers with a single 2×2 max pooling layer in between as shown in Fig 2. Each of the first two stages consists of two consecutive convolutional layers while each of the middle two stages consists of five consecutive convolutional layers. The last stage includes four consecutive convolutional layers. A convolutional layer used in this architecture consists of a number of filters (i.e. 64, 128, 256, 512, and 512 for each of the five stages respectively) where each filter has a size of 3×3×3. The output of the five stages of convolutional layers was then fed to a set of two consecutive, fully connected layers with 4096 neurons each. The last fully connected layer has 3 neurons for non-linear classification. A dropout layer was also applied to the outputs of the first two fully connected layers where 50% of the outputs were dropped to further minimize overfitting and improve the robustness of the architecture.

Obviously, the proposed CNN architecture can be seen as a modified version of the VGG-19 where two convolutional and rectified linear units were added to the middle two stages while the last fully connected layer with 1000 neurons was replaced with a three-neuron layer. The parameters of the proposed architecture were initialized using the pretrained weights and biases of the original VGG-19 architecture, which had been previously and successfully trained and tested on the ImageNet dataset [18]. In addition, the initial parameters of the additional proposed convolutional and final fully connected layers were set to the identity operation. The full architecture was further trained and validated for DR classification, and staging, where the parameters of the whole model were fine-tuned and updated on the Kaggle dataset [17].

### C. Evaluation metrics

In this sub-section, evaluation metrics used to validate and measure the performance of the proposed network are described. In this study, both 5-fold and 10-fold cross validation methods were considered. In 5-fold cross validation, the dataset was split into 5 groups of 920 images while, in 10-fold cross validation, 10 groups of 460 images were considered. Validation accuracy is then defined as follows:

$$Accuracy=\frac{TP+TN}{TP+FP+TN+FN}$$ (6)

where *TP* and *FP* of a specific category *C* are the true positive (i.e. when an image belonging to *C* was correctly classified as *C*) and false positive (i.e. when an image not belonging to *C* was falsely classified as *C*), respectively. Also, *TN* and *FN* are the true negative (i.e. when an image not belonging to *C* was not classified as *C*) and false negative (i.e. when an image belonging to *C* was classified as non *C*), respectively. Further, sensitivity and specificity are defined as follows:

$$Sensitivity=\frac{TP}{TP+FN}$$ (7)

$$Specificity=\frac{TN}{TN+FP}$$ (8)

The overall classifier sensitivity and specificity can be estimated by averaging individual sensitivities and specificities for each class respectively. Moreover, final sensitivity and specificity were averaged over the 5 folds and 10 folds. Confusion matrix was used to describe the performance of the proposed classifier. A confusion matrix is represented by a table with each row containing the counts of images with certain predicted labels and each column including the counts of images with certain actual labels. Entries of this table can be defined as the number of images that share a specific predicated as well as actual labels. Further, the receiver operating characteristic (ROC) curve was plotted to determine the ability of the classifier to successfully distinguish between various categories. It describes the relationship between the true positive rate (sensitivity) and the false positive rate (1 –specificity) at various threshold settings with the area under the ROC curve (AUC) measuring the separability of the classifier. The higher the AUC, the more capable the classifier to differentiate between different classes of the disease.

Although the validation accuracy is considered an acceptable performance measure, it may not be fully describing the performance of the proposed architecture since the model was trained on a dataset with unequal category sizes. Quadratic weighted Kappa score is an another approach that evaluates the performance of the classifier and measure the agreement between two raters; predicted labels, and ground truth labels [19]. The score ranges from -1 which represents a total disagreement between predictions and ground truth to 1 which represents a complete agreement between both labels. The score can also be 0 if the agreement between labels took place by chance. The quadratic weighted Kappa score can then be calculated in five steps as follows:

1. Calculate and then normalize the confusion matrix (*C*).
2. Create the weights matrix W where more weight is assigned to the predictions of higher deviation from actual labels. Weights are given using the following formula:
  $$w(i,j)=\frac{{\left(i-j\right)}^{2}}{{\left(L-1\right)}^{2}}$$ (9)
3. Create and then normalize the histogram of both actual labels vector and predicted labels vector.
4. Calculate and normalize the outer product (*P*) of the two histograms.
5. Calculate the quadratic weighted Kappa (*K*) as follows:
  $$K=1-\frac{\sum_{i=0}^{L}\sum_{j=0}^{L}w(i,j)c(i,j)}{\sum_{i=0}^{L}\sum_{j=0}^{L}w(i,j)p(i,j)}$$ (10)

## III. Experimental results

The proposed model described in the previous section was trained on the Kaggle dataset [17] for 15 epochs. The learning rate was set at 10^−3^. Prior to training the model, the dataset was divided into 5 folds and 10 folds in order to validate the model using 5-fold and 10-fold cross validation methods respectively. Each fold was further split into batches of 57 fundus images in order to reduce the computational complexity of the training process by deploying the SGD rather than a gradient descent over the entire training set. Training accuracies were found to be 91% (respectively, 92%) for 5-fold (respectively, 10-fold) cross-validation of the model.

Tables 2 and 3 show the confusion matrix when a 5-fold, and 10-fold cross validation were deployed, respectively. From both tables, we noticed that confusion matrix values at the top right and bottom left corners are very low which may indicate the ability of the classifier to discriminate between subjects belonging to classes with greater differences in label.

**Table 2:** Confusion matrix for the proposed model (5-fold cross validation).

	No DR	Moderate DR	Severe DR
**No DR**	351	9	0
**Moderate DR**	10	234	7
**Severe DR**	0	78	231

**Table 3:** Confusion matrix for the proposed model (10-fold cross validation).

	No DR	Moderate DR	Severe DR
**No DR**	174	8	3
**Moderate DR**	1	122	6
**Severe DR**	0	27	119

Table 4 shows the validation accuracy, sensitivity and specificity of the proposed architecture, and related work [4] [5] [7] [9]. It is quite obvious that proposed architecture outperforms the related work in terms of the validation accuracy with almost 14% enhancement over [7] [9], 7% and 3% increase with respect to [4] and [5], respectively when a 10-fold cross validation was considered. Further, the sensitivity as well as the specificity of the proposed model is elevated compared with [4] [5] [7].

**Table 4:** Validation accuracies, sensitivities and specificities of the proposed CNN and related work.

	Proposed CNN Architecture		Pratt et al. [7]	Dekhil et al. [9]	Acharya et al. [4]	Acharya et al. [5]
	5-Fold Cross Validation	10-Fold Cross Validation				
**Accuracy**	88%	89%	75%	75%	82%	85.9%
**Sensitivity**	87%	89%	30%	N/A	82%	82%
**Specificity**	94%	95%	95%	N/A	88%	86%

**Fig 3:**
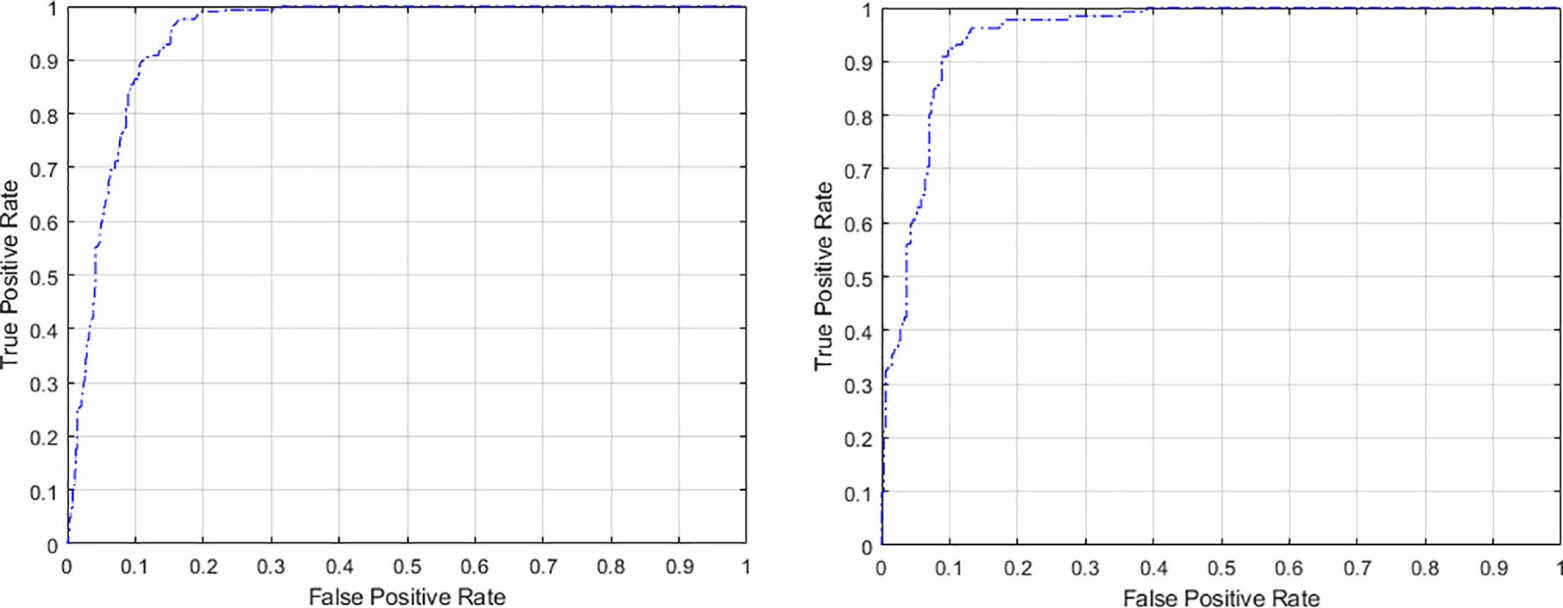
ROC curve for the proposed model in case of (a) 5-fold cross validation (b) 10-fold cross validation.

Fig 3 shows the ROC curve for the proposed model when both 5-fold and 10-fold cross validation were used at a specific threshold setting. It is clear that at 0.1 false positive rate, a high true positive rate of almost 0.9 was achieved. Further, AUC of 0.95, and 0.91 were calculated when 5-fold and 10-fold cross validation were deployed, respectively, indicating a promising use for the proposed classifier to separate between DR stages based on fundus images.

To account for the bias of the model towards relatively large-sized category (i.e. non DR) where the model is more sensitive towards the aforementioned category as compared to the moderate and severe DR categories, the quadratic weighted Kappa score was calculated for both the 5-fold and 10-fold cross validation of the model, since this score will give partial credit to misclassification with less deviation from the ground truth. Shown in Table 5, the quadratic weighted Kappa score of the proposed model was found to be almost 0.92 when a 10-fold cross validation was used surpassing the performance of the model introduced by [9].

**Table 5:** Area Under the Curve (AUC) and quadratic weighted Kappa score when 5-fold and 10-fold cross validation is used.

	5-Fold Cross Validation	10-Fold Cross Validation
**AUC**	0.95	0.91
**Quadratic Weighted Kappa Score**	0.91	0.92

**Fig 4:**
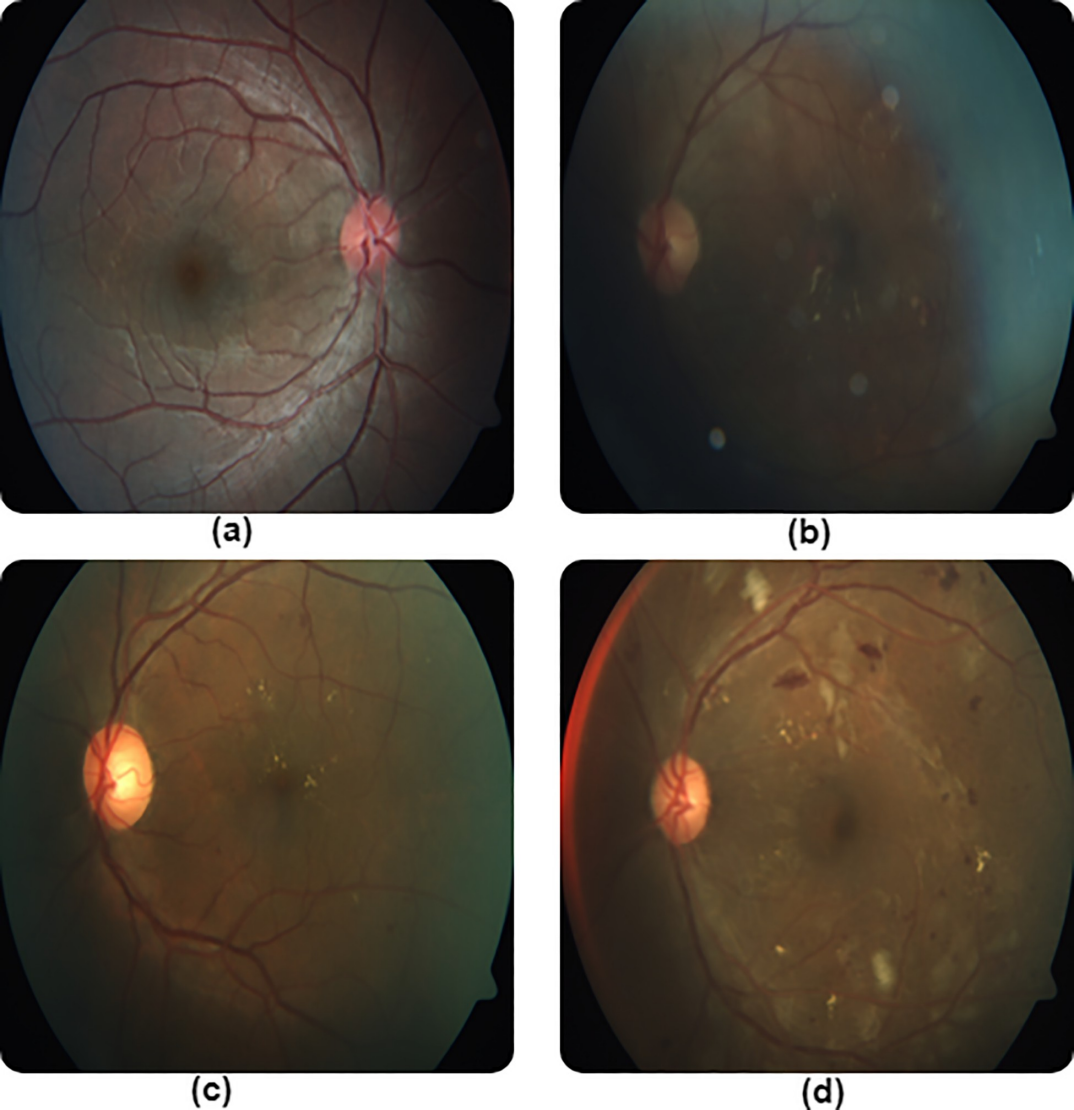
Examples of misclassified fundus images by the proposed architecture. (a) Ground Truth “0” Predicted “1”. (b) Ground Truth “1” Predicted “2” (c) Ground Truth “1” Predicted “0” (d) Ground Truth “2” Predicted “1”.

We have also provided examples of the worst case predictions where images were misclassified by the proposed classifier. Fig 4 shows the aforementioned examples. As shown in Fig 4, we may probably attribute the inability of the proposed model to classify the images to lighting effects in the captured images and poor contrast levels.

## IV. Discussion

In this paper, the feasibility of a deep convolutional neural network to accurately diagnose and classify DR using fundus images was demonstrated. The proposed approach resulted in a higher diagnostic accuracy, sensitivity, and specificity when compared to other CNN-based and SVM-based techniques published in literature [4] [5] [7] [9]. Significantly, the proposed method can also grade DR with a high degree of accuracy in addition to its high diagnostic accuracy.

Automated grading of DR solves two major problems in clinical ophthalmology. First, it can be applied to instant grading of telemedicine fundus images. Many patients in rural areas in the developed world and throughout the developing world do not have easy access to subspecialty ophthalmic care. Remotely based fundus cameras have been the key tool in ophthalmic telemedicine, taking fundus photographs of diabetics and then sending these digitally to ophthalmologists located elsewhere. However, these images must still be interpreted by an expert and the final diagnosis communicated in a delayed fashion to the patient. Our system can in theory be applied at the time of photography, providing the patient and local healthcare provider with an instant diagnosis. This is a substantial labor- and time-saving tool.

Second, grading of DR made in the clinic by the examining physician are not always accurate. Inter-observer variability for grading DR varies from 0.62–0.87 in different studies [20, 21]. This system provides a highly accurate and consistent diagnosis and grading of DR, a significant improvement over the human error and variability inherent in human diagnosis. The proposed approach eliminates these limitations, and enables both diagnosis and quantification of the degree of DR, expanding care and access. Further, disease progression or the effectiveness of treatment can be objectively compared from one visit to another, which can enable physicians to optimize medical therapy.

The limitations of the proposed approach are: (1) it can only classify the diabetic retinopathy into three categories where both mild and moderate NPDR are represented by one group, and severe NPDR and PDR are combined in another group; (2) neural network models in general are considered as black boxes which make it difficult to interpret the results or the features extracted while SVM methods [4] [5] extract handcrafted features which can be helpful for medical specialists to identify the biomarkers of DR; and (3) deep learning techniques including CNN are susceptible to overfitting. Overfitting arises when the model is trained using a limited dataset and fails when the trained model is applied on a new data set. A limited dataset for training will not allow the model to extract the appropriate features that help the model for successfully classifying new data. To minimize overfitting and to provide an unbiased evaluation on the available limited dataset, class-specific data augmentation as well as 5-fold and 10-fold cross validation were used. To further improve the accuracy of the proposed approach, we will train images of different modalities using the proposed CNN model in the future. Furthermore, we will further deploy capsule networks, recently introduced by Geoffrey Hinton, to classify fundus images [16]. Capsule networks provide further details on the presence and the pose of features using primary and routing capsules (i.e. a group of neurons whose task is to extract a specific feature) with less training data. It has proven its superiority over traditional CNN when used to classify the popular MNIST handwritten digit images database.

## V. Conclusion

In the current study, a deep CNN architecture composed of 18 convolutional layers and 3 fully connected layers was introduced to classify and stage DR, where the subjects were classified into no DR, moderate DR, and severe DR. Overall, 4,600 fundus images were generated from the original Kaggle dataset [17] using a class-specific data augmentation technique, and used to train and test the proposed network using 5-fold and 10-fold cross validation.

A quadratic weighted Kappa score of 0.92 and a validation accuracy of 89% were achieved, providing an improvement over the results obtained by deep CNN architectures [7] [9] by almost 14% as well as SVM Based classifiers [4] [5] with an accuracy improvement of 7% and 3% respectively when a 10-fold cross validation was used. Further, the proposed model surpassed all the-state-of-the-art architectures [4] [5] [7] [9] with respect to sensitivity and specificity. With our proposed approach, ophthalmologists may accurately and objectively detect and stage DR in a timely manner and possibly monitor its progression without the need for the traditional subjective physical assessment that may lack sensitivity or precision.
